# The Joint Action Effect on Memory as a Social Phenomenon: The Role of Cued Attention and Psychological Distance

**DOI:** 10.3389/fpsyg.2017.01697

**Published:** 2017-10-05

**Authors:** Ullrich Wagner, Anna Giesen, Judith Knausenberger, Gerald Echterhoff

**Affiliations:** Department of Psychology, University of Münster, Münster, Germany

**Keywords:** joint action, social memory, incidental encoding, psychological distance

## Abstract

In contrast to individual tasks, a specific social setting is created when two partners work together on a task. How does such a social setting affect memory for task-related information? We addressed this issue in a distributed joint-action paradigm, where two team partners respond to different types of information within the same task. Previous work has shown that joint action in such a task enhances memory for items that are relevant to the partner’s task but not to the own task. By removing critical, non-social confounds, we wanted to pinpoint the social nature of this selective memory advantage. Specifically, we created joint task conditions in which participants were aware of the shared nature of the concurrent task but could not perceive sensory cues to the other’s responses. For a differentiated analysis of the social parameters, we also varied the distance between partners. We found that the joint action effect emerged even without sensory cues from the partner, and it declined with increasing distance between partners. These results support the notion that the joint-action effect on memory is in its core driven by the experience of social co-presence, and does not simply emerge as a by-product of partner-generated sensory cues.

## Introduction

A distinct characteristic of the human species is the extraordinary level of cooperation between individuals, even between genetically unrelated conspecifics. Evolutionary accounts explain this human “hypersociality” (Pinker, 2010) by a selection pressure toward joint and coordinated action in early human societies. Because social action conditions social cognition and vice versa, the emergence of socially oriented action tendencies presumably also shaped the underlying human cognitive system in such a way that the processing of information relevant to jointly performed activities receives priority over the processing of other information (Smith and Semin, 2004; Mesoudi et al., 2006; Shteynberg, 2014). Within the cognitive system, memory is a particularly important function. This is because memory represents the critical mechanism for the continued availability of socially relevant information beyond the current situation, which affords preparatory adaptation to social interactions in the future. However, little is known so far on how joint task performance affects memory formation.

The present research addresses this issue in a distributed task setting, in which two team partners are asked to respond to different types of information embedded within the same speeded response task. Our experimental study builds on a paradigm by Eskenazi et al. (2013), who found that participants exhibited enhanced memory for items that were relevant to the partner’s part of the task but not to their own part. By removing critical, non-social confounds, we aimed at pinpointing the social nature of the memory advantage for partner-relevant items. Specifically, we created joint task conditions in which participants were clearly aware of the shared nature of the concurrent task but could not perceive sensory cues to the other’s responses. For a differentiated analysis of the social parameters of partner-oriented memory enhancement, we also varied the distance between partners during task performance.

### Task Sharing Effects on Memory for Partner-Relevant Information

To understand how joint action affects individual memory, Eskenazi et al. (2013) employed an experimental paradigm to investigate how joint task performance affects memory encoding of the material presented during the task. This paradigm draws on previous studies on joint action tasks, which have shown by means of response time measures that individuals attend to the partner’s tasks even when they perform independent tasks alongside each other (Sebanz et al., 2003, 2005). The study from Eskenazi et al. (2013) extends this work by showing that such involuntary attention toward stimuli relevant to the partner’s task also entails enhanced memory encoding of these stimuli. This finding suggests that joint action effects occur not only on-line, i.e., during the execution of a task itself, but also with consequences on the longer run.

The task used in the experimental paradigm from Eskenazi et al. (2013) was a simple word categorization task performed by two participants alongside each other. Exemplars of three different word categories (animals, fruit/vegetable, or household objects) were successively presented on a computer monitor. Participants were invited in pairs, and each of the partners had the task to react to only one of the three word categories by a key press whenever a word of this category appeared on the computer monitor, with different word categories assigned to the two participants. As in previous studies on response conflicts in joint action tasks (Sebanz et al., 2003, 2005), the condition of joint task performance, where the two participants worked on the task together sitting next to each other, was compared with a condition of individual task performance (with each person performing the task alone) to identify the role of the social context that is created by joint task performance.

Importantly, in a subsequent surprise memory test, participants had to write down (in individual tests) as many words as possible from all the words they had encountered during the previous word categorization task, regardless of word category and encoding condition (joint vs. individual). The critical finding was that significantly more words from the category assigned to the partner were remembered under joint than individual task performance, an effect that was not found for the word category assigned to none of the two partners, nor for the word category that elicited own action. Also, within the condition of joint action, more words were recalled from the word category assigned to the partner than from the word category assigned to no-one. Hence, despite being equally irrelevant to own task performance, stimuli perceived as relevant to a partner in a joint action task receive priority in memory encoding over stimuli perceived as irrelevant to the partner. Importantly, this effect occurs involuntarily, because during encoding of the stimuli, i.e., during performance of the word categorization task, participants do not know that memory for the words will be tested subsequently (incidental encoding). Thus, the results indeed point to incidental mnemonic effects specifically for socially relevant stimuli in the context of joint task performance.

However, the experimental procedure from Eskenazi et al. (2013) leaves open a straightforward, non-social explanation, which invokes attention-grabbing effects of sensory cues. As described, the two participants performed the task together in the joint action condition sitting next to each other at the same computer monitor and using the same keyboard. Under such circumstances, the condition of joint task performance differs from individual task performance not only by providing a social context in which two partners act simultaneously on the same task, but also by the presence of additional perceptual cues due to the actions of the partner. Specifically, participants can visually and auditorily perceive the partner’s key presses that occur in response to words from the category assigned to the partner. These key presses provide perceptual cues in this word category that are not present in the condition of individual task performance. Deeper encoding of the words from the partner’s word category under joint task performance might therefore have resulted simply from externally driven attention toward perceptual cues elicited by the partner’s actions, irrespective of the social vs. non-social nature of the source of these cues.

According to this cued attention account, enhanced memory would simply result from different perceptual encoding conditions elicited by the presence of the actions of another person, but not necessarily from the social nature that is created under conditions of joint task performance (see Dolk et al., 2011, for a similar discussion about the social nature of a common on-line effect of joint action, the so-called “Social Simon Effect”). Accordingly, only if the effect can be shown to occur also in the absence of the confounding perceptual factors, the cued attention account can be ruled out. To do so, one has to create conditions where joint and individual task performance do not differ with regard to the opportunity to see or hear partner-generated cues.

The primary goal of the present study was to examine the genuinely social nature of the joint action effect on memory encoding by removing confounds resulting from perceptual cues. For this purpose, we modified the paradigm by Eskenazi et al. (2013) to have two participants work at two different computers during joint task performance. While working in the same room, as in the original procedure, participants were separated by a partition wall and wore soundproof headphones during the task. This modification eliminated both visual and auditory cues from the partner’s actions. Joint-task effects on the concurrent processing of partner-relevant information have been amply demonstrated (Sebanz et al., 2003, 2005), also without concurrent visual or auditory cues to the co-actor’s responses (e.g., Vlainic et al., 2010). Thus, assuming the joint-task effect on subsequent memory as an effect that is essentially driven by the social context created by joint task performance, we expected to find such an effect also in this modified task setting, because the social characteristics remained the same as in the original task setting.

The elimination of visual perception would also rule out the possibility that enhanced memory for partner-relevant items results from motor simulation during the observation of the partner’s task-related actions. It has been found that the observation of actions performed by another person can induce false memories of having performed the other’s actions, an effect referred to as observation inflation (Lindner et al., 2010, 2016). In the joint-action paradigm, observing the partner’s actions (i.e., responses to assigned items) may create, perhaps via motor simulation, mnemonic representations of having performed the action. During recall, the memory advantage found for self-assigned items may thus also extend to memory for items assigned to the partner. Finding the joint-task effect also in the modified task setting would suggest that such observation-induced processes are not critical.

This would also be in line with recent results from Elekes et al. (2016), who applied Eskenazi et al.’s (2013) paradigm to a task that did not require overt motor responses. (Participants had to silently count the number of words from their assigned word category.) These authors focused on the difference between the three kinds of word assignment *within* a joint task setting, leaving out a control condition of individual task performance. Therefore, joint and individual task performance could not be compared. Still, the fact that the authors found better memory for partner-assigned words than for task-irrelevant words even in a non-motor task speaks against a motor simulation account and also suggests social rather than perceptual factors as primary determinants of memory effects of joint task performance. However, in the absence of a control condition of individual task performance, the effect of the social setting of joint task performance cannot be evaluated. To confirm the critical joint encoding effect as an interaction between item assignment and encoding context (joint vs. individual) as in the original study from Eskenazi et al. (2013), both factors have to be manipulated, and this is what we do in the present study.

### The Role of Psychological Distance

In addition, we wanted to examine the social parameters of the effect more directly, specifically, the role of psychological distance. The concept of psychological distance has been introduced and explicated by Liberman and Trope (2014). In brief, psychological distance refers to any circumstances in which something receives psychological relevance to a person although it is outside the direct “here and now” experience of the person in the current situation. Psychological distance can be created by spatial or physical distance, but also on other dimensions, that is, on the time dimension (event in the future or past rather than event in the present situation), on the dimension of hypotheticality (subjectively improbable or only imagined event rather than actually occurring event), or on the social relationship dimension (event not occurring to me but to another person, who is familiar or unfamiliar or subjectively similar or dissimilar to me). Interestingly, research has found that psychological distance perceived on the different dimensions is positively correlated. That is, activities or events that are distal on one dimension were judged as distal also on the other dimensions (Fiedler et al., 2012).

Originally, the concept of psychological distance was developed specifically within the context of construal level theory (Liberman and Trope, 2014), where it has been identified as the primary determinant of cognitive abstraction processes, while it has not received much attention so far outside this specific theoretical framework. However, as a “basic dimension of meaning in a way similar to valence” (Liberman and Trope, 2014, p. 365), psychological distance is likely to affect a variety of other fundamental cognitive and emotional processes, including those related to memory formation. Indeed, psychological distance has recently been identified as a critical determinant of affective processing of socially shared experiences (Wagner et al., 2015; Boothby et al., 2016), and initial findings also point to an influence of psychological distance on mnemonic processes (Smith and Trope, 2006; Fukura et al., 2013).

Here, we specifically manipulated psychological distance to examine the social parameters of the joint-action effect in memory. The underlying idea is that information attended to by a partner is perceived as more relevant to possible further interaction when the partner is close (vs. distant). This is because interaction is more likely and feasible the closer one is to the other person. As outlined in the beginning, models of socially conditioned cognition (Smith and Semin, 2004; Mesoudi et al., 2006; Shteynberg, 2014) assume that the processing of information relevant to social interaction and jointly performed activities is prioritized over the processing of other information. Accordingly, the enhanced encoding of partner-relevant stimuli may depend on the perceived distance between the two partners.

In the joint-action paradigm, the manipulation of psychological distance can be straightforwardly implemented via physical distance, that is, by seating the participants at different locations (Guagnano et al., 2010; Atmaca et al., 2011; Welsh et al., 2013). Our modified version of the task, where the two partners are separated and perform the task at different computers, creates more distance compared to the original procedure by Eskenazi et al. (2013). If enhanced memory for partner-relevant words are not simply an effect of visual or auditory cues but driven by social factors, the effect should decrease with increased distance between the partners.

To test this assumed connection between psychological distance and the extent of the joint encoding effect as a monotonous relationship, at least three conditions of psychological distance are needed. We therefore divided our total sample into three experimental groups, differing in spatial distance between the two partners involved in joint task performance. In a first group, which replicated the procedure from Eskenazi et al. (2013), the joint action task was performed by both partners at the same computer. In this condition physical distance was minimal. In the second group, the two partners sat in the same room but were separated by a partition wall, as described above. In this modified same-room group condition, physical distance was increased and both visual and acoustic cues during joint task performance were eliminated. Finally, in a third group, the spatial distance between the two partners was further increased by having the two participants perform the joint task in different rooms (different-rooms condition). As in the modified same-room condition, the partner’s actions cannot be seen or heard in this third condition, but psychological distance is additionally augmented because performing the task in different rooms also attenuates the sense of co-presence.

### Predictions of the Present Study

To sum up, we predicted that enhanced memory for partner-relevant items in the joint vs. individual task conditions would be found in the full sample. The extent of the effect should monotonously decrease with psychological distance. That is, it should be strongest when the two partners of a pair perform the joint task sitting next to each other at the same computer (replication condition of low distance) and weakest when they perform it in different rooms (different-rooms condition, creating high distance). In the modified same-room condition, where the partners are physically and perceptually separated from each other within the same room (medium distance), the effect should fall in between. We expected that the effect *per se* should be obtained not only in the replication condition. Because we suspected that the effect is not simply driven by the perceptual consequences of the partner’s actions, it should be found also in the modified same-room condition (although to a numerically somewhat lesser extent according to the previous hypothesis). In the different-rooms condition alone, where the perceived relevance of the partner’s responses is lowest, the effect might not be strong enough to emerge *per se*, depending on the gradient of effect attenuation across the three distance levels. Currently, there are no data regarding memory formation available on this issue, but studies on motor performance in joint action tasks point to two different possibilities in this different-room condition. On the one hand, it is possible that the joint-action effect on memory formation still occurs under such conditions where the presence of the co-actor during task performance is only imagined, as has been found for motor performance in joint action tasks (Tsai et al., 2008; Atmaca et al., 2011), but on the other hand the perceived distance of the partner may have attenuated the social nature of the context to such an extent that the effect is not substantial any more (Guagnano et al., 2010).

## Materials and Methods

### Participants and Design

Seventy-five students from the University of Münster (Germany) participated in the experiment in exchange for course credit or monetary compensation. They were randomly assigned to one of the three experimental groups in a 3 (distance: same computer, modified same-room, different-rooms) × 3 (assigned item category: self, other, none) × 2 (social encoding context: joint vs. individual) mixed design, with the first independent variable varied between participants.

The study was carried out in accordance with the recommendations of the local ethics committee at the University of Münster with written informed consent from all subjects. All subjects gave written informed consent in accordance with the Declaration of Helsinki. The protocal was approved by the ethics committee at the Department of Psychology at the University of Münster.

The sample size was determined on the basis of the size of the joint encoding effect in the original study by Eskenazi et al. (2013), i.e., $\eta_{p}^{2}$ = 0.19 for the interaction between item category and social encoding context and, more specifically, an effect size of *d*_z_ = 0.63 for the critical comparison between joint and individual encoding for the partner-assigned items. Power analyses performed by G^∗^Power (Faul et al., 2007) indicated that such an effect in pairwise within-subjects comparisons could be detected with samples of *n* = 18 for a single experimental group, assuming a Type I error of 0.05 (one-tailed test) and a power of 0.80. Because the effect may be smaller in our modified conditions, the sample size was further increased by a third beyond this threshold.

Participants were recruited individually, but took part as pairs of partners who were invited for the same date. If only one participant could be recruited for a given experimental date, a confederate from the lab served as the real participant’s partner (which was the case for 16 participants). Confederates’ data were not included in the data analysis. Post-experimental questioning made sure that none of the pairs coincidentally consisted of two persons who were already well acquainted with one another.

### Materials and Procedure

The task was based on the procedure from Eskenazi et al. (2013). First, the two participants of each pair were introduced to each other by their forenames and were seated next to each other to sign the informed consent and to read task instructions about the subsequent categorization task. In this categorization task, each participant was assigned one of three word categories (animals, fruit/vegetable, and household objects) and was instructed to respond as fast as possible by pressing a specified key whenever a word of this assigned category appeared on the computer monitor, and to do nothing whenever a word from another category appeared, avoiding mistakes (Go/NoGo task). For example, one participant always had to respond to animals (but not to household objects or fruits/vegetables), while the other participant always had to respond to household objects (but not to animals or fruits/vegetables). Each participant performed this task of pressing a key in reaction to one specific word category once alone (individual condition) and once simultaneously together with the partner in the pair (joint condition). Thus, from a given participant’s perspective, there were three word categories according to task assignment: “Self” (words to which oneself always had to respond), “Other” (words to which oneself never responded, but the partner did during joint task performance), and “None” (words that never required any response from either partner).

The task instructions for each participant were the same during individual and joint conditions, i.e., to respond as fast and accurately as possible to words from the “Self” category (which always remained the same for both individual and joint task performance), so that any performance differences between joint and individual conditions can be attributed to involuntary influences of the awareness that another participant was simultaneously involved in the task during joint task performance (with a different word category assignment). The order of the individual vs. joint condition of task performance, as well as the assignment of specific word categories to “Self,” “Other,” and “None” conditions were counterbalanced across subjects. (In conditions of individual task performance, the word categories “Other” and “None” were equivalent, because the partner was not involved. Still, following Eskenazi et al. (2013), the same three word category labels as in the respective joint task performance conditions were also used here, so that each of the three word categories “Self,” “Other,” and “None” in the joint task condition had words from the same semantic category as a corresponding control in the individual task condition.)

The material consisted of 144 German nouns (48 words denoting animals, 48 words denoting fruits/vegetables, and 48 words denoting household objects), subdivided into two parallel sets of words (each containing 24 nouns from each category) for use in the joint and individual task condition. The two word sets were matched overall and within each of the three word categories for word length and word frequency. Assignment of the two word sets to the joint and individual task conditions was balanced across subjects. Furthermore, as an additional measure to reduce any possibly biasing effects of single words within the lists, rough semantic matching was ensured within each word category. For example, a bird in the word category “animals” in one word set was paralleled with another bird in the other word set. The categorization task was implemented by E-Prime 2 software. Two keys on the computer keyboard were used to make responses, one for each participant in a pair. Stimuli were presented in random order. Each trial started with a fixation cross of 500 ms, followed by a stimulus word presented for either 1,500 ms or 3,000 ms. (The factor of stimulus duration did not influence any experimental factor here and is therefore not further considered in the following.)

Each task block started with a short practice run (using additional words not included in the word lists for the main run) to give participants the opportunity to become familiar with the specific procedures. Critically, in addition to Eskenazi et al.’s (2013) original study, to investigate the role of perceptual cues from the partner and of social distance during joint encoding, participants were assigned to three different groups that differed in these factors. First, in the same-computer group (*n* = 24), equivalent to the situation in Eskenazi et al.’s (2013) original study, the two participants of each pair sat next to each other during joint task performance, looking at the same computer monitor and using the same keyboard to give their responses.

Second, in the modified same-room group (*n* = 26), participants sat in the same room, but they performed the task at separate computers and keyboards and were prevented from seeing and from hearing each other by visual partitions installed between the computers and by soundproof headphones that participants wore during task performance. (Participants were told that they were given the headphones to ensure their optimal concentration during the task.)

Finally, in the different-rooms group (*n* = 25), participants sat at different computers in different rooms during joint task performance. Thus, in addition to the lack of perceptual cues from the partner, physical distance between the partners was maximal in this condition. Thus, the social component of joint task performance was reduced to an abstract level in this condition, i.e., joint and individual task performance were absolutely equivalent apart from the information given by the experimenter regarding the involvement of the other person during task performance.

To ensure that the presence of the partner could not be simply “forgotten” during task performance under such abstract social conditions, the program was individualized and included a feedback screen after each word presentation, indicating which would have been the correct response in this trial, i.e., a key press by oneself, a key press by the partner, or no key press. This feedback was given in a personalized manner by using the forenames of the participants and their respective partner. That is, in the end of each trial, the feedback “keypress [forename of participant],” “keypress [forename of participant’s partner],” or “no keypress” appeared on the screen. (Note that this feedback did not give information on the actual performance of the partner, but only repeated the assignment of word categories that was already known from the general instructions.) This kind of feedback was presented in all three experimental groups, and it was given under both joint and individual conditions of task performance (and also in the practice runs), where in the individual condition only the two feedback alternatives “keypress [forename of participant]” or “no keypress” were applicable.

In the individual task condition, the two participants were always separated and performed the task in different rooms. As noted, each participant’s instructed task was the same for both the individual and the joint condition (keypress to words from one of the three semantic word categories). Instructions in the second block pointed out that the task from the previous block was basically performed again, the difference being only (if the second block was the joint block) that the partner would now be also involved by pressing a key in response to words from another specified semantic category or (if the second block was the individual block) that the partner would no longer be involved now and would instead perform a different task in a separate room. A new practice run was always performed at the beginning of the second run to familiarize participants with the new condition.

After participants had completed both task blocks (individual and joint), followed by a short unrelated distraction task, the surprise free-recall memory test was performed. In this test, participants were asked to write down on an empty sheet of paper as many words from the previous categorization task (regardless of word category) as they could. This memory task was performed individually by all participants (in separate rooms, so that the partner was not present during this retrieval phase). Five minutes were given for this memory task, which was abundant time for all participants. The critical dependent variable was the percentage of correctly recalled words in each experimental condition, and in particular the joint encoding effect as indicated by the difference in memory performance between joint and individual encoding. Words recalled from practice runs were not included in the analysis.

At the end of the session, we assessed participants’ experience of social co-presence. Specifically, participants were asked in two separate questions about their subjective awareness of the other participant during joint and individual task performance (on 10-point scales, ranging from 1 = “not at all aware” to 10 = “highly aware”). Previous findings have shown that subjective awareness of a non-visible partner during joint task performance in an affective picture viewing task is associated with a positive shift in emotional reactions toward pictures viewed simultaneously with the partner in comparison to those viewed alone (Wagner et al., 2015). Following Wagner et al.’s (2015) findings, we expected that our experimental manipulation of spatial distance should be reflected in an enhancement of subjective awareness of the partner in joint as compared to individual task performance. As in Wagner et al. (2015), we subtracted scores for the individual task condition from scores for the joint task condition, resulting in a relative score of experienced co-presence.

The alpha level in all analyses was set to 0.05. For the specific, one-sided hypotheses based on previous findings, one-tailed tests were performed. All other tests were two-tailed.

## Results

**Table 1** shows the percentage of correctly recalled words and the joint encoding effects for the different experimental conditions for the sample as a whole and separately for the three experimental groups. Results of a 2 (joint vs. individual encoding context as a within-subjects factor) × 2 (assigned item category: “Self,” “Other,” “None” as a within-subjects factor) × 3 (Psychological distance: same computer [low distance], modified same room [medium distance], different rooms [high distance], representing a between-subjects factor) analysis of variance (ANOVA) performed on the percentage of correctly recalled words showed a significant main effect of word category [*F*(2,144) = 52.94, *p* < 0.001], with self-assigned words being generally better remembered than the other two word categories, as also found by Eskenazi et al. (2013), reflecting the typical self-reference effect in memory (Symons and Johnson, 1997).

**Table 1:** Mean percentages (with standard deviations in parentheses) of correctly recalled words and extent of the joint encoding effect (difference joint – individual encoding; right column) as a function of distance during task performance (same computer/modified same room, i.e., perceptually separated within the same room/different rooms), assigned item category (self/other/none), and social encoding context (joint vs. individual action).

	Assigned item category	Joint action (Social encoding context)	Individual action (Non-social encoding context)	Joint encoding effect
Total sample	*Self*	18.94 (9.35)	21.94 (11.14)	-3.00 (12.47)
	**Other**	**13.50 (10.04)**	**9.56 (6.84)**	** 3.94 (10.47)**
	None	10.06 (6.85)	11.22 (8.05)	-1.16 (8.54)
Same computer (low distance)	*Self*	18.92 (7.96)	20.14 (9.09)	-1.22 (10.09)
	**Other**	**14.93 (11.46)**	**8.51 (7.11)**	** 6.42 (11.46)**
	None	7.81 (6.54)	10.07 (7.67)	-2.26 (8.11)
Modified same room (medium distance)	*Self*	21.15 (9.42)	24.84 (14.70)	-3.69 (15.20)
	**Other**	**14.26 (10.08)**	**9.94 (8.09)**	** 4.33 (11.57)**
	None	11.22 (6.75)	12.34 (8.70)	-1.12 (9.25)
Different rooms (high distance)	*Self*	16.67 (10.27)	20.67 (8.02)	-4.00 (11.69)
	**Other**	**11.33 (8.46)**	**10.17 (5.11)**	** 1.17 (8.11)**
	None	11.00 (7.00)	11.17 (7.86)	-0.17 (8.20)

*Note: Positive joint encoding effects selectively occur for other-assigned words (shown in bold).*

More importantly, a significant interaction between word category and encoding condition [*F*(1,144) = 9.10, *p* < 0.001] indicated a replication of the main finding of Eskenazi et al.’s (2013) original study for our sample as a whole, i.e., a selective encoding advantage of joint encoding as compared to individual encoding for other-assigned words (words to which the partner had to react), but not for words to which either oneself or nobody had to react. This selective advantage was confirmed by *t*-test comparisons that revealed, as expected, higher memory performance after joint than individual encoding for other-assigned words [*t*(74) = 3.23, *p* = 0.001, one-tailed]. In contrast, memory performance for words assigned to self or to no-one was numerically even lower after joint than individual encoding, with this opposing effect reaching significance for self-assigned words, *t*(74) = -3.23, *p* = 0.041, two-tailed. The three-way interaction did not reach significance, *F*(4,144) = 0.86, *p* = 0.49. Thus, there was no evidence that the joint-encoding effect differed significantly between the three experimental groups, so that all groups appeared to contribute to the overall pattern of differences between joint and individual task performance. Inspection of **Table 1** indeed confirms for all three groups a numerically positive joint encoding effect for other-assigned words, accompanied by a numerically negative joint encoding effect in the other two word categories.

However, the pattern also shows the assumed gradient in the advantage of joint encoding for the other-assigned words depending across different levels of psychological distance, i.e., with the largest advantage in the situation where the partners are sitting next to each other at the same computer while performing the task together (in the same-computer group) and the smallest advantage in the situation where they are sitting in separate rooms while performing the task together (in the different-rooms group). This gradient was statistically confirmed by a Jonckheere–Terpstra trend test for a decrease of the extent of the joint encoding effect for the other-assigned words from the same-computer group to the different-rooms group (*J* = 1120.5; *z* = 1.799; *p* = 0.036, one-tailed). No respective trends were found for word categories assigned to the self (*p* = 0.52, two-tailed) or to nobody (*p* = 0.37, two-tailed).

The three groups did not differ in overall memory performance, and additional control analyses showed that the order of joint vs. individual task conditions had no influence on memory performance overall or in interaction with other factors (all *p*s > 0.16).

When the three groups were analyzed separately based on the a priori hypotheses, the critical two-way interaction between word category and joint vs. individual encoding and the respective pairwise comparison of joint vs. individual encoding for other-assigned words remained significant in the same-computer group [interaction *F*(2,46) = 4.785, *p* = 0.021; joint vs. individual encoding for other-assigned words: *t*(23) = 2.746, *p* = 0.006, one-tailed] and in the modified same-room group [interaction *F*(2,50) = 3.928, *p* = 0.029; joint vs. individual encoding for other-assigned words: *t*(25) = 1.906, *p* = 0.034, one-tailed], but not in the different-rooms group [interaction *F*(2,48) = 1.851, *p* = 0.170; joint vs. individual encoding for other-assigned words: *t*(24) = 0.719, *p* = 0.240, one-tailed]. Effects sizes for the joint encoding effect for the other-assigned words with respective 95% confidence intervals (CI) were for same-computer group, *d*_z_ = 0.561, CI [0.124; 0.987], for modified same-room group, *d*_z_ = 0.374, CI [-0.028; 0.768], and for different-rooms group, *d*_z_ = 0.144, CI [-0.252; 0.536].

To check the psychological efficacy of the spatial distance manipulation, we analyzed participants’ awareness of the partner’s co-presence. The corresponding scores differed between the three experimental groups, *F*(2,73) = 3.18, *p* = 0.047. The enhancement of experienced co-presence was more pronounced in the same-computer group (*M* = 4.79, *SD* = 2.30) compared to the different-rooms group (*M* = 3.04, *SD* = 2.48; *p* = 0.04), with the modified same-room group (*M* = 3.65, *SD* = 2.59) falling in between these values. A Jonckheere–Terpstra trend test confirmed that the experience of co-presence declined monotonically with increasing psychological distance (*J* = 1183; *z* = 2.41; *p* = 0.016).

At postexperimental questioning, two participants expressed doubt that the other person had been acting as a real partner in the joint task condition. When data from these participants were excluded, the pattern of results remained the same in all statistical analyses.

## Discussion

Our findings shed new light on the social dimension of the joint-task effect on memory for partner-relevant stimuli (Eskenazi et al., 2013). The effect was replicated with the original procedure, where the two participants performed their parts of the task at the same computer. Importantly, it also occurred when the two partners were divided by a partition wall during joint task performance and wore soundproof headphones, preventing access to all visual and auditory cues from the partner. However, the extent of the effect was numerically and statistically less pronounced with this modified procedure. This is in line with our second hypothesis of a monotonous relationship between psychological distance between the partners and the extent of the effect, because the physical separation of the partners also enhances psychological distance between them. Consistent with this hypothesis, the effect was further diminished and lacked statistical significance *per se* in a third experimental group in which psychological distance was further enhanced by joint task performance taking place with the partners sitting not only at separate computers (within the same room), but even in different rooms.

A formal statistical test across the three groups confirmed the monotonic decrease of the specific effect of joint vs. individual task performance for partner-relevant words (but not for words that are self-relevant or not relevant to either partner) with increasing psychological distance between the partners. Notably, however, there was no significant three-way interaction between the joint vs. individual task performance, word category, and the group factor representing psychological distance. This indicates that at least the general pattern was the same in all three groups and all of them obviously contributed to some degree to the clear confirmation of the basic Eskenazi et al. (2013) finding, represented by a strong joint/individual × word type interaction, in our sample as a whole across the three groups, although the lack of significance in the three-way interaction might also simply be attributed to a lack of power and could have been obtained with a bigger sample size.

On the whole, our results allow several important conclusions regarding the nature of the joint encoding effect that Eskenazi et al. (2013) have described. First, the effect is robust and replicable. As a mnemonic phenomenon, it confirms, together with Eskenazi et al.’s (2013) original findings, that joint action cannot only affect individual behavior online, during task execution (Sebanz et al., 2003, 2005), but can also exert effects that remain present in an individual’s mind on the longer run, beyond the immediate situation of joint vs. individual action. Furthermore, our results show that the joint-action effect on memory is in its core genuinely social, i.e., it is essentially the result of the social meaning attributed to partner-relevant stimuli in the context of joint task performance, and not simply a by-product of additional attention-driving perceptual cues associated with the partner’s actions (as assumed by the cued attention account; see Dolk et al., 2011, for a similar discussion on the inherently social nature of the immediate effects of joint action on reaction times during task performance). The cued attention account can be excluded here, because the effect was also obtained when the availability of such additional perceptual cues was prevented by a partition wall placed between the partners. Likewise, in line with previous findings from Elekes et al. (2016), an explanation based on motor simulation of the partner’s actions (Lindner et al., 2010) can be ruled out by this procedure, because there was no visual access to the partner’s motor responses.

Finally, putting the results of all three experimental groups together, psychological distance between the partners appears to be a critical determinant of the effect, influencing subjective awareness of each other in the partners’ mind. We varied distance between the partners across the three groups and found a declining trend of the specific joint encoding effect with increasing distance between the interaction partners, in parallel with a concomitant decline in the extent of subjective awareness of the partner’s co-presence induced by joint task performance. The original situation from the Eskenazi et al. (2013) study, where the two partners act together at the same computer and can perceive each other’s actions, representing the condition with lowest distance (accompanied by the highest level of partner awareness), yielded the strongest joint encoding effect. On the other hand, the situation where the two partners performed the joint action task while sitting in different rooms, representing the condition of highest distance (accompanied by the lowest level of partner awareness), yielded the least pronounced joint encoding effect (with lack of statistical significance in this condition *per se*).

Based on these findings, the fact that the extent of the effect was strongest under the original conditions of Eskenazi et al. (2013), where the two partners performed the joint task side by side at the same computer, may be accounted for by two different (although mutually not exclusive) explanations. On the one hand, perceptual cues from the partner, although not absolutely necessary, may add independently, at least to some degree, to the in principle socially driven effect via direct sensory channels that are guiding attentional resources. On the other hand, it is also possible that these perceptual cues subjectively add to the low psychological distance situation in this condition that concomitantly also creates the highest level of awareness of the partner. In this latter case, they would exert indirect effects by forming a part of those features that make a situation socially relevant on a subjective level. In any case, the present study shows that the existence of such perceptual cues alone is not sufficient to explain the occurrence of the joint encoding effect, and that this effect is instead driven by inherently social parameters of the joint encoding situation.

Following Eskenazi et al. (2013), the present study investigated effects of joint encoding specifically in the context of a joint action task. However, because our findings speak against motor simulation as a critical underlying mechanism, we would not claim that the joint encoding effect for partner-relevant stimuli is necessarily tied to joint action, i.e., a task that requires overt motor responses from the two involved partners (occurring in a more or less coordinated manner). Rather, we would assume on the basis of our findings that effects of joint encoding can emerge in any (motor or non-motor) task as long as joint encoding enhances social relevance of certain stimuli. Results from Shteynberg (2010, Exp. 2), who used a very different paradigm, are in line with this assumption. In this online experiment, participants were tested in (virtual) groups and were led to feel similar (vs. dissimilar) to each other in a “minimal group” manipulation (Tajfel and Turner, 1979). In the beginning of the experiment, each participant had to choose an avatar symbolically representing himself or herself during the session. Immediately after having chosen a color, they were informed that all the other players had chosen the same color (similarity condition) vs. a different color (dissimilarity condition). Later, participants were presented with a word list, followed by a recognition memory test for the words. Memory performance in this test was better when participants believed similar others (vs. dissimilar others) to perform the same task simultaneously. Interestingly, the similarity/dissimilarity manipulation can be regarded as a manipulation of psychological distance (lower psychological distance for similar than for dissimilar partners) as in our present study. However, there was no control condition of individual encoding, so we do not know the effect of joint encoding *per se*. But the results at least do show that in this task, in the absence of any motor component, joint encoding enhanced memory performance when the partners involved perceived each other as being similar to each other (presumably increasing the perceived social relevance of the jointly attended stimuli) than when they did not.

The follow-up study to Eskenazi et al. (2013) by Elekes et al. (2016) also included a non-motor task derived from the same paradigm. In that task version, participants counted silently the number of words from their assigned category instead of pressing a key when a word from this category appeared. The results indicated enhanced memory for other-relevant words than for non-relevant words under joint encoding conditions even in this task version, where no overt motor response was required. However, as described earlier, conditions of individual encoding were not included in this study, so that the focus was only on the difference between conditions within joint encoding. Thus, it was impossible to analyze differences between joint and individual encoding in different word categories, as in the present study and in the original Eskenazi et al. (2013) study. However, inspection of our data in **Table 1** shows that the same monotonous gradient across psychological distance that we observed for the joint encoding effect for other-relevant words is also visible when, as in Elekes et al.’s (2016) study, the difference between the partner-relevant words and the words that are non-relevant to either partner *within* the condition of joint task performance is considered.

Indeed, when a statistical trend test is performed on these data (means of differences: same-computer group: 7.12%; modified same-room group: 3.04%; different-rooms group: 0.33%), the monotonous decline with increasing psychological distance is likewise confirmed (*J* = 1145.5; *z* = 2.046; *p* = 0.041, two-tailed). As we used a motor task as in Eskenazi et al.’s (2013) original study, it is interesting to compare these data to the pattern in the motor task condition in Elekes et al.’s (2016) study, which was performed in one experiment at the same computer (representing our lowest distance condition) and in another experiment in different rooms (representing our highest distance condition). The effect on the difference in encoding between partner-relevant words and task-irrelevant words turned out to be strong and highly significant in the same-computer condition, but small and non-significant in the different-rooms condition, a pattern that parallels our present findings.

Nevertheless, despite obviously converging results when only word categories within joint encoding conditions are considered, in our view the comparison between joint and individual task performance on which we focus in the present study remains more informative. Only in this comparison the difference between encoding in a social vs. a non-social context of task performance becomes directly manifest. Future research should further investigate mnemonic effects of joint task performance with different kinds of motor and non-motor tasks in experimental paradigms that include the direct comparison between joint and individual encoding conditions.

Regarding our sample as a whole, there is one interesting difference to the original pattern of results from Eskenazi et al. (2013). Although our results clearly replicate their joint/individual × word type interaction and confirm the stronger memory encoding for joint vs. individual encoding specifically for the partner-relevant words, we additionally found – in contrast to Eskenazi et al. (2013) – a concomitant *detrimental* effect of joint as compared to individual encoding for the self-relevant words. That is, encoding of self-relevant words was on the whole even worse under joint than individual encoding conditions. Although this effect was much less pronounced than the positive effect of joint encoding on the partner-relevant words, this result opens the possibility that in our study the positive effect on partner-relevant words occurred at the expense of an opposite effect on self-relevant words. This, however, was not the case because there was no significant negative correlation between the two effects (i.e., between the joint-individual difference in the “self” and the “other” condition). In fact, the correlation was even numerically positive (*r* = 0.139, *p* = 0.235), so that, if any, a relative advantage of joint vs. individual encoding in one word category was associated with the same advantage also in the other word category.

In our view, the relative inferiority of joint over individual encoding for self-relevant words may be best interpreted as an attenuating effect on the self-focus under conditions of joint task performance. Such self-focus, needed to actively concentrate one’s mind on the words relevant to one’s own motor responding (a self-related attention focus that also leads to the overall best memory encoding for these self-relevant words, i.e., the self-reference effect in memory; Symons and Johnson, 1997), may be more difficult to maintain under conditions of joint than individual encoding because of the additional (involuntary) social information processing that takes place during joint task performance. Interestingly, as indicated by our correlation analyses, the positive mnemonic effect of this involuntary social information processing with regard to partner-relevant stimuli occurs independently of the opposite effect of joint task performance on the mnemonic consequences of the active voluntary attentional focus on self-relevant stimuli, and the former effect is overall stronger than the latter one.

In sum, the present study confirms the memory effects of joint vs. individual encoding revealed by Eskenazi et al. (2013) in a joint action task as an inherently social phenomenon whose extent is related to the psychological distance between the partners during joint task performance. Together with other findings (Shteynberg, 2010; Elekes et al., 2016), these data point to a natural and automatic tendency of the human cognitive system to prioritize information of social relevance in the process of memory encoding. Future research should further investigate the generalizability of these results to other tasks of joint encoding (with and without coordinated motor action) and to other manipulations of psychological distance (e.g., manipulations that more directly affect the perceived social relationship between the two partners), and should further specify the exact mechanisms driving these effects.

## Author Contributions

UW and GE designed the study. UW, AG, and JK performed the study. UW and GE analyzed data. UW, AG, JK, and GE wrote manuscript.

## Conflict of Interest Statement

The authors declare that the research was conducted in the absence of any commercial or financial relationships that could be construed as a potential conflict of interest.
